# Chikungunya virus adapts to tiger mosquito *via *evolutionary convergence: a sign of things to come?

**DOI:** 10.1186/1743-422X-5-33

**Published:** 2008-02-27

**Authors:** Xavier de Lamballerie, Eric Leroy, Rémi N Charrel, Konstantin Ttsetsarkin, Stephen Higgs, Ernest A Gould

**Affiliations:** 1 Institut de Recherche pour le Développement UMR190/Unité des Virus Emergents, Université de la Méditerranée, Marseille, France; 2 Institut de Recherche pour le Développement UMR190/CIRMF, Franceville, Gabon; 3 Department of Pathology, University of Texas Medical Branch, Galveston, Texas, USA

## Abstract

Since 2004, several million indigenous cases of Chikungunya virus disease occurred in Africa, the Indian Ocean, India, Asia and, recently, Europe. The virus, usually transmitted by *Aedes aegypti *mosquitoes, has now repeatedly been associated with a new vector, *Ae. Albopictus*. Analysis of full-length viral sequences reveals three independent events of virus exposure to *Ae. Albopictus*, each followed by the acquisition of a single adaptive mutation providing selective advantage for transmission by this mosquito. This disconcerting and current unique example of "evolutionary convergence" occurring in nature illustrates rapid pathogen adaptation to ecological perturbation, driven directly as a consequence of human activities.

## Findings

**Figure 1:**
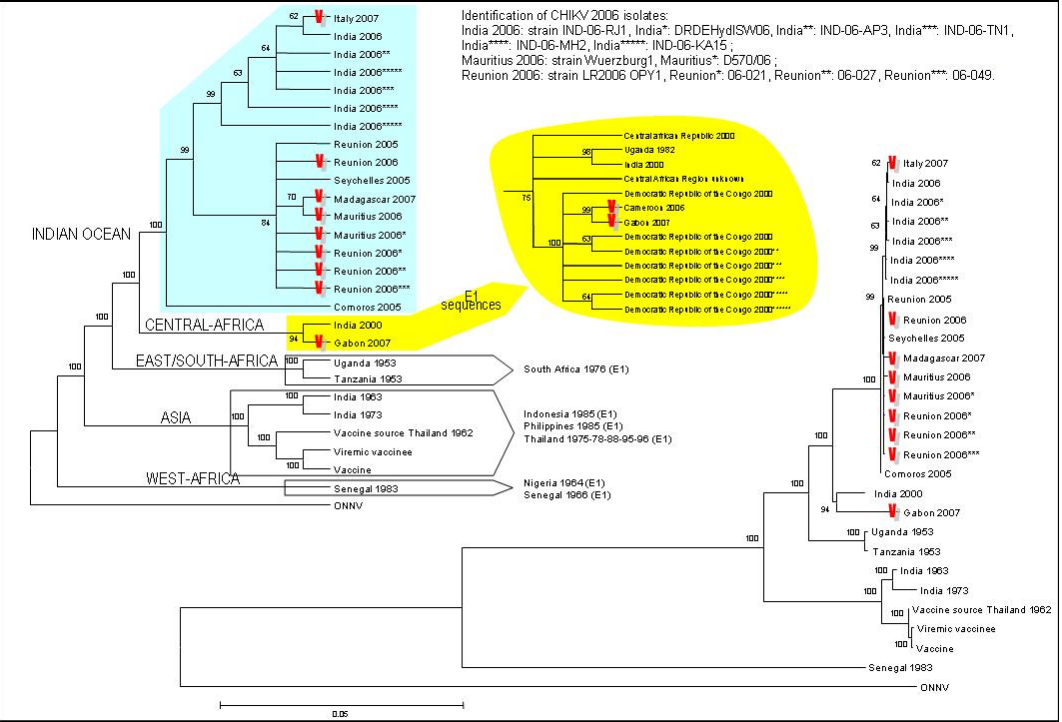
**Chikungunya virus dispersal and evolution**. Phylogenetic trees were produced using alignments of complete or nearly-complete Chikungunya virus nucleotide sequences, from which the E1 226 codon was removed. Bootstrap resampling values are indicated at the main branches. Strains with the A226V mutation are indicated. In the tree on the right, horizontal bars are proportional to genetic distances. The tree on the left shows only the topology of the reconstruction. Branches supported by a bootstrap <60 are collapsed. Colours that identify the different lineages are the same as in figure 2. Central African strains that have been assigned to a given lineage based only on partial sequencing of the E1 gene are indicated in the exploded yellow bubble (a phylogenetic branch reconstructed from E1 sequences is shown). Isolates in the East-South-Africa, Asia and West-Africa lineages, which have been characterised only in the E1 gene are indicated.

**Figure 2:**
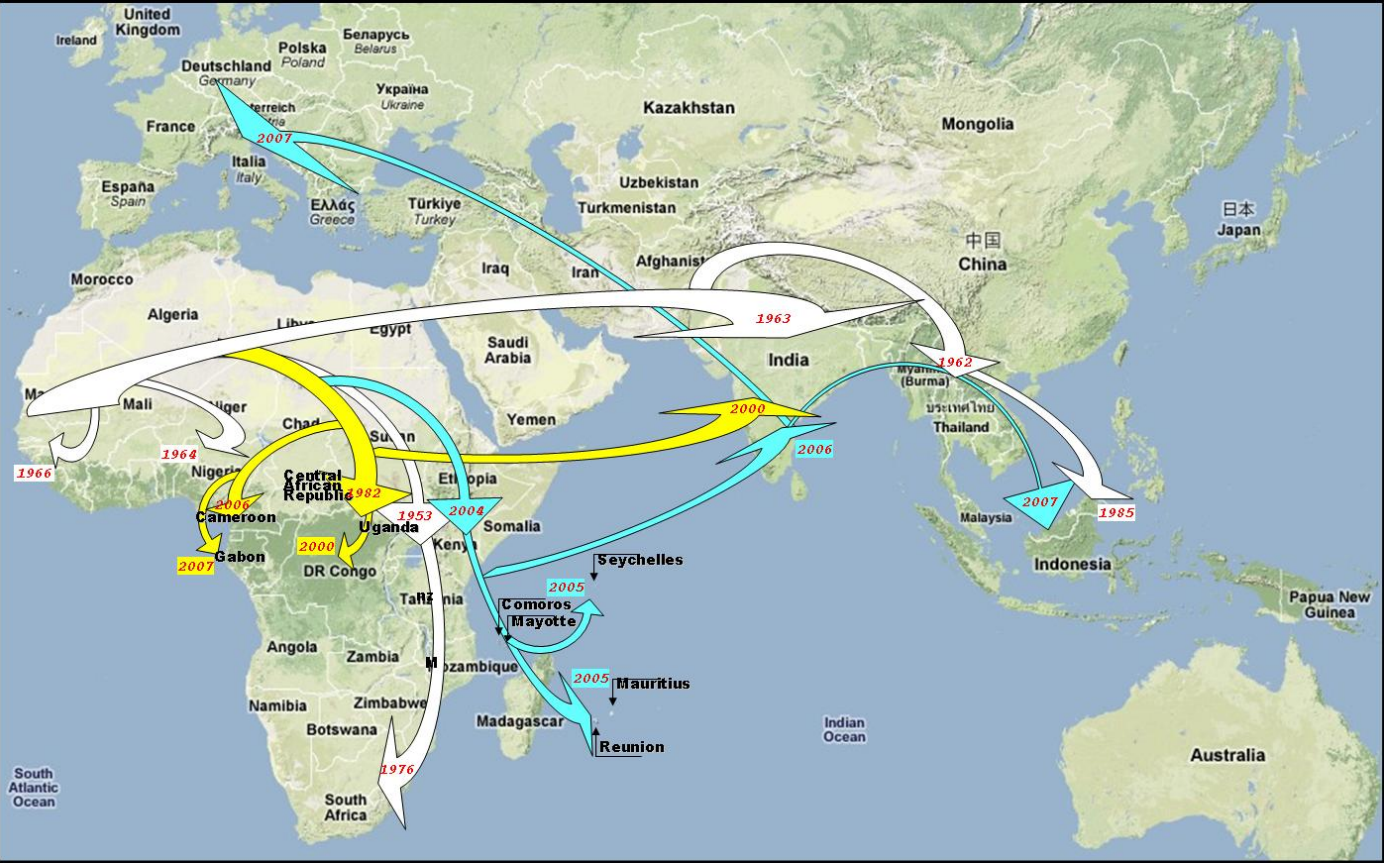
Predicted dispersal pattern of Chikungunya virus from Africa to the Indian Ocean and Europe during the past 20 to 50 years.

Mosquito-transmitted Chikungunya virus (CHIKV) is responsible for explosive outbreaks of febrile arthralgia in humans [1,2]. Several evolutionary lineages have been identified (fig-1) corresponding to, Western-Africa, Asia, East/South-Africa and Central-Africa [3]. Phylogenetic analyses of full-length genomes reveal that CHIKV is readily transported by infected travellers to distant locations where it generates new outbreaks (fig-2). This propensity for dispersal and emergence in remote ecological environments illustrates the adaptability of the virus, in particular to new vector populations.

Until recently, during human outbreaks, the principal identified vector of CHIKV was *Ae. aegypti*. However, CHIKV has been recently associated with an alternative vector, *Ae. Albopictus *(the "Asian Tiger Mosquito"), which has spread in areas previously occupied predominantly by *Ae. aegypti*, and dispersed globally via commercial transportation of, for example scrap car tyres [4].

During the 2004 epidemic in Kenya and the subsequent outbreaks, when CHIKV was introduced into Comoros and Seychelles, CHIKV was transmitted by *Ae. aegypti*. Previous studies showed that *Ae. Aegypti*-associated CHIKV isolates from Comoros and Seychelles, as well as early isolates from other islands in this region, had an Alanine residue at position 226 of the E1 gene [5]. However, when the virus reached Reunion and Mauritius Islands, it met different ecological environments in which *Ae. aegypti *is absent or scarce and *Ae. albopictus *predominates. Within one year, a new mutation (A226V, *ie *a Valine residue at position 226) was identified in some CHIKV samples [5]. The virus also reached Madagascar and Mayotte, where both *Ae. aegypti *and *Ae. albopictus *are common. The A226V mutation was identified in all sequenced 2006 Mayotte isolates [5] and in a recent 2007 Madagascan isolate (this study). This suggests that this mutation is associated with adaptation to *Ae. Albopictus *and, indeed, we have recently shown that it improves virus replication and transmission efficiency in this mosquito [6]. To conclude, in all Indian Ocean islands where *Ae. albopictus *is present, the A226V adaptive mutation was observed 1 or 2 years after the introduction of CHIKV. Whether this mutation was acquired several times independently or if an "*Ae. albopictus*-adapted" strain evolved in one island and then dispersed to neighbouring islands is unknown.

The situation is different in India. Our phylogenetic analyses suggest that CHIKV originating from East-Africa or Comoros was introduced into India in 2006 (fig-1&2). In 2007, an infected traveller from India arrived in Italy and caused more than 200 indigenous cases of chikungunya. The Italian strain ITA07-RA1 (GenBank_EU244823) has the A226V mutation, acquired in Italy (where *Ae. albopictus *is present) or, more probably, in India (where both *Ae. aegypti *and *albopictus *are present). Since 2006 Indian isolates originate from an ancestor with an Alanine at position 226 (fig-1), the A226V mutation must have been acquired independently from the identical mutation of the Indian Ocean isolates. Additional evidence supports the case for independent mutations. Chikungunya outbreaks were observed in Cameroon (2006) and Gabon (2007) [2], where *Ae. albopictus *has displaced *Ae. aegypti*. CHIKV strains from both outbreaks originate from the Central-African lineage (ie, are distinct from Indian/Indian Ocean isolates from the same period), but, in contrast to original Central-African strains (transmitted by *Ae. aegypti*) both the Cameroon (Chik_Cam_7079, GenBank_EF051584) and Gabon (this study) isolates have the A226V mutation. This implies an independent adaptive mutation in response to a similar requirement of transmission by *Ae. albopictus*.

It is extremely rare for this phenomenon, known as "evolutionary convergence", to be observed in nature. In virology, convergent mutations have been reported under the extreme selective pressure of antiviral therapy during the treatment of acute (eg neuraminidase mutations of influenza virus) or chronic (eg reverse-transcriptase/protease mutations of HIV) viral diseases. Our results demonstrate that the selective pressure exerted on CHIKV through the constraint of having to replicate in a new vector, is similar to that cited for antiviral therapy. Since the dispersal of *Ae. albopictus *from Asia to Europe and the Americas is largely the result of human commercial activities, the adaptation of CHIKV to *Ae. albopictus *provides a fascinating demonstration of how viruses can readily circumvent the impact of human interference on the ecosystem. Our observations also have very serious implications for future emerging arboviruses that infect humans. *Aedes albopictus*, which has dispersed into central Africa is also becoming widespread in Europe and North-America. Thus, CHIKV, and possibly other tropical arboviruses, have the potential to invade more northerly geographic regions.

## Abbreviations

CHIKV: Chikungunya virus.

## Competing interests

The authors declare that they have no competing interests.

## Authors' contributions

XdL led and coordinated the project and the manuscript redaction, realised phylogenetic analysis. EL isolated and characterised Gabon strains (viral genomics), was involved in data analysis and manuscript redaction. RNC isolated and characterised Madagascar strains (viral genomics), was involved in data analysis and manuscript redaction. KT characterised Reunion strains, made substantial contribution to analysis, have been involved in manuscript redaction. SH made substantial contribution to analysis and interpretation of data, was involved in manuscript redaction. EAG substantially contributed to phylogenetic analysis and interpretation of data, was involved in manuscript redaction. All authors approved final version of the manuscript.
